# 

**Published:** 1887-05

**Authors:** 


CONTENTS.

Vol. 34 No. 5.

| | PAGE. |
|---|---|
| The Occult Forces | 97 |
| Injurious and Adulterated Beverages | 102 |
| Water, Its Agency in Therapeutics | 106 |
| Dr. R. C. Fisher's Fasting Cure | 108 |
| Strange Admonition of Death | 111 |
| Food Adulterations | 112 |
| Molecules and Atoms | 113 |
| Some Forebodings of Incipient Insanity | 114 |
| Book Notices | 115 |
| Household | 116 |
| Miscellaneous | 118 |

INDEX TO ADVERTISEMENTS OF HALF A PAGE AND OVER.

| | PAGE. |
|---|---|
| Winchester's Hypophosphate | I |
| Hollow Suppositories | II |
| Celerina | III |
| Lactopeptine | IV |
| Castoria | IV |
| Horsford's Acid Phosphate | V |
| Bovinine | VII |
| Ten Valuable Books Free | VIII |
| Lactated Food | 2d page cover |
| Crystalline Phosphate, | 3d page cover |
| Goodyear Rubber Co. | 3d page cover |

